# Beyond “implementation strategies”: classifying the full range of strategies used in implementation science and practice

**DOI:** 10.1186/s13012-017-0657-x

**Published:** 2017-11-03

**Authors:** Jennifer Leeman, Sarah A. Birken, Byron J. Powell, Catherine Rohweder, Christopher M. Shea

**Affiliations:** 1 School of Nursing, University of North Carolina at Chapel Hill, CB #7460, Chapel Hill, NC 27599 USA; 2 Department of Health Policy and Management, Gillings School of Global Public Health, University of North Carolina at Chapel Hill, Chapel Hill, NC 27599 USA; 3 Center for Health Promotion and Disease Prevention, University of North Carolina at Chapel Hill, Chapel Hill, NC 27599 USA

**Keywords:** Implementation strategies, Dissemination, Scale-up, Interactive Systems Framework, Capacity-building

## Abstract

**Background:**

Strategies are central to the National Institutes of Health’s definition of implementation research as “the study of strategies to integrate evidence-based interventions into specific settings.” Multiple scholars have proposed lists of the strategies used in implementation research and practice, which they increasingly are classifying under the single term “implementation strategies.” We contend that classifying all strategies under a single term leads to confusion, impedes synthesis across studies, and limits advancement of the full range of strategies of importance to implementation. To address this concern, we offer a system for classifying implementation strategies that builds on Proctor and colleagues’ (2013) reporting guidelines, which recommend that authors not only name and define their implementation strategies but also specify who enacted the strategy (i.e., the actor) and the level and determinants that were targeted (i.e., the action targets).

**Main body:**

We build on Wandersman and colleagues’ Interactive Systems Framework to distinguish strategies based on whether they are enacted by actors functioning as part of a Delivery, Support, or Synthesis and Translation System. We build on Damschroder and colleague’s Consolidated Framework for Implementation Research to distinguish the levels that strategies target (intervention, inner setting, outer setting, individual, and process). We then draw on numerous resources to identify determinants, which are conceptualized as modifiable factors that prevent or enable the adoption and implementation of evidence-based interventions. Identifying actors and targets resulted in five conceptually distinct classes of implementation strategies: dissemination, implementation process, integration, capacity-building, and scale-up. In our descriptions of each class, we identify the level of the Interactive System Framework at which the strategy is enacted (actors), level and determinants targeted (action targets), and outcomes used to assess strategy effectiveness. We illustrate how each class would apply to efforts to improve colorectal cancer screening rates in Federally Qualified Health Centers.

**Conclusions:**

Structuring strategies into classes will aid reporting of implementation research findings, alignment of strategies with relevant theories, synthesis of findings across studies, and identification of potential gaps in current strategy listings. Organizing strategies into classes also will assist users in locating the strategies that best match their needs.

## Background

Strategies are central to the National Institutes of Health’s (NIH’s) definition of implementation research as “the study of strategies to integrate evidence-based interventions into specific settings” [1]. Multiple papers have been written proposing frameworks and lists to describe the strategies used in implementation research and practice [2, 3]. In a recent review, Lokker et al. [4] identified 23 models, frameworks, and taxonomies for classifying the type of strategies used “to promote and integrate evidence into practice in healthcare.” These strategies increasingly are being classified under the single term “implementation strategies” [2]. We contend that classifying all strategies under a single term may constrain efforts to synthesize findings across tests of strategy effectiveness and limit advancement of the full range of strategies of importance to implementation science and practice.

The ability to synthesize findings across studies is essential to building the evidence base for what, how, and when implementation strategies work to improve which implementation outcomes. Despite the valuable work that has been done to name and define strategies, efforts to synthesize findings across studies continue to be challenging [4]. Names and definitions do not adequately differentiate who enacted the strategies or for what purpose, resulting in findings that are not “readily comparable” or amendable to the synthesis needed to build the evidence base for their use [5].

We illustrate the problem with an example from our research. Two authors (JL, CR) are collaborating on a study testing “implementation strategies” that practice facilitators (e.g., coaches) provide to primary care clinic staff to build the staffs’ capacity to use “implementation strategies” to integrate cancer screening evidence-based interventions (EBIs) into their practice setting. The broad use of the term “implementation strategy” creates confusion for our team because it does not distinguish what the practice facilitators are doing to support implementation from what primary care staffs are doing to integrate EBIs into routine practice. We contend that these distinctions are important when selecting and reporting strategies for use in research and practice because the evidence base and, to a large extent, the underlying theory, differ for strategies used to support implementation (e.g., [6, 7]) as compared to strategies used to integrate screening EBIs into practice (e.g., [8, 9]).

The use of the single term “implementation strategy” also increases the risk that some of the strategies essential to implementation science will be overlooked. Those using the term “implementation strategy” tend to define it in relation to the implementation of a specific intervention or guideline. Powell et al. [2], for example, define “implementation strategies” as “methods or techniques used to enhance the adoption, implementation, and sustainability of a clinical program or practice.” Similarly, Mazza et al. [3] define an “implementation strategy” as a “purposeful procedure to achieve clinical practice compliance with a guideline recommendation.” These definitions suggest that implementation begins with a predefined “program or practice” or “guideline” and have the potential to perpetuate the field’s historic focus on a “push” model of implementation that begins with the identification of a specific evidence-based intervention (EBI) that is then “pushed” into practice [10]. Many scholars have called for more research that applies a “pull” model, with the goal of building practice-level capacity to select, adapt, and implement the EBIs they need to address locally identified needs [10, 11]. We contend that to achieve this goal, implementation science needs to invest more in strategies that build practice-level capacity to prioritize areas in need of improvement and to select (i.e., pull) from the menus of available EBIs. The creation, promotion, and distribution of these EBI menus require dissemination strategies—yet another set of strategies of importance to implementation.

To address these challenges (among others), Proctor et al. [5] recommended that authors not only name and define their implementation strategies but also describe how they operationalized the strategy in their study. Specifically, they recommended specifying who enacted the strategy (i.e., the actor), the level and determinants that were targeted (action targets), and the intended implementation outcomes. Clearly delineating how strategies are operationalized is critical to reporting findings in ways that are amenable to synthesis. Using a more consistent approach to operationalizing strategies also has potential to foreground the full range of strategies used in implementation science.

Building on the recommendations of Proctor et al. [5], we propose a system for classifying implementation strategies based on the strategies’ actor and action targets. (Table 1 provides an overview of definitions for these and other terms used in this paper.) Structuring strategies into classes will aid in aligning strategies with relevant theories (i.e., those that pertain to related determinants), synthesizing findings across studies, and identifying potential gaps in current strategy listings. Organizing strategies into classes also will assist users in locating the strategies that best match their needs. We are not suggesting a new taxonomy but rather a system for classifying implementation strategies.

**Table 1:** Key terms and their definitions

Evidence-based intervention (EBI)	“Programs, practices, principles, procedures, products, pills, and policies” that have been found to be effective at improving health behaviors, health outcomes, or health-related environments [21]
Actor	Who enacts the strategy [5]
Delivery system actors	Individuals, teams, and systems that adopt and integrate EBIs into practice [12]
Support system actors	Individuals, teams, and systems that build delivery systems’ general and EBI-specific capacity to adopt and integrate EBIs [12]
Synthesis and translation systems	Organizations that identify, translate, and disseminate EBIs [12]
Implementation strategies (action)	“Methods or techniques used to enhance the adoption, implementation, and sustainability” of EBIs [2]
Action target	What the strategy intends to change [5]
Levels	The level the strategy targets (intervention, individuals, inner setting, outer setting, processes) [18]
Determinants	The modifiable factors the strategy intends to change to overcome barriers and activate facilitators of EBI adoption and implementation [15]

### Classifying strategies according to who enacts them (the actor)

To differentiate categories of actors, we drew on the three systems described in the Interactive Systems Framework (ISF) for dissemination and implementation [12]. *Delivery system* actors include the individuals and teams who adopt and integrate EBIs into their practice settings. They include those working in public health departments, hospitals, clinics, and community-based coalitions and organizations, among others. *Support system* actors promote and support EBI adoption and implementation with a focus on building delivery systems’ “general capacity” to adopt and implement EBIs as well as their “EBI-specific capacity,” in other words, their capacity to adopt and implement specific EBIs [12]. Support system actors often are external to the setting where implementation will occur and in the USA include, for example, Area Health Education Centers [13] or State Health Departments [14], both of which employ staff to provide technical assistance (i.e., quality improvement coaches or practice facilitators) to those working in delivery system to promote and support EBI adoption and integration. Support system actors also may function within delivery systems, particularly larger systems that employ quality improvement coaches and other staff who then provide support to the staff who are adopting and integrating EBIs into practice. *Synthesis and translation system* actors identify, translate, and disseminate EBIs and include, for example, the US Preventive Services Taskforce, the Cochrane Collaboration, and a host of other organizations that synthesize, translate, and disseminate EBIs in print and electronic formats.

### Classifying strategies by linking them to action targets

Action targets include both the determinant and the level that an implementation strategy targets. The determinants targeted may include any modifiable factor that prevents or enables EBI adoption and implementation [15]. Several recently published frameworks contend that the determinants’ strategy targets are central to defining both interventions and implementation strategies. In their framework for knowledge translation interventions, Colquhoun et al. [16] identified “what they aim to change” as one of four defining components. (The other three components are the strategy or technique, causal mechanism, and mode of delivery). In creating a taxonomy of behavior change interventions, Kok et al. [17] also argued for linking behavior change methods to a modifiable determinant of behavior change. Strategies may target determinants at multiple levels including the EBI (e.g., its complexity), inner setting (characteristics of the setting into which the EBI is implemented, e.g., leadership engagement), outer setting (characteristics of the wider socio-political context, e.g., public policy), individuals (characteristics of those intended to adopt and implement the EBI; e.g., motivation, ability), and process (the presence and nature of activities involved in EBI adoption and implementation, e.g., planning, evaluating) [18]. Numerous taxonomies and lists are available that detail determinants derived from reviews of the literature and theory and often organized by level (e.g., [15, 19, 20]).

## Classifying implementation strategies

**Fig. 1:**
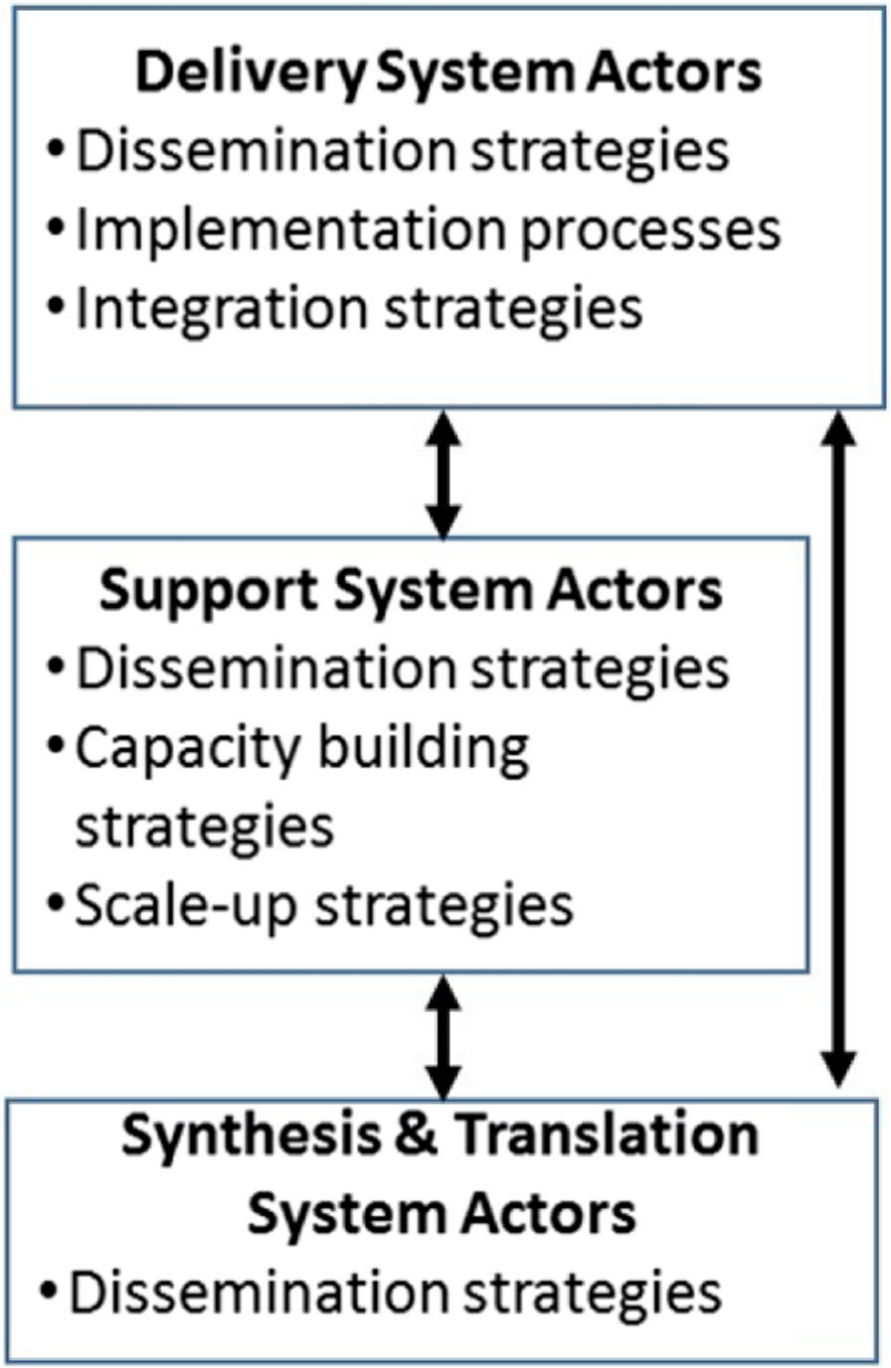
Classes of implementation strategies organized within the Interactive System Framework [12]. The bi-directional arrows represent the importance of communication across levels

As further described below, identifying actors and action targets resulted in five conceptually distinct classes of strategies: dissemination, implementation process, integration, capacity-building, and scale-up (See Fig. 1 and Table 1). In our descriptions of each class, we identify the ISF system actors that enacted the strategy (actors), levels and determinants targeted (action targets), and outcomes used to assess strategy effectiveness. We illustrate how each class would apply to strategies to improve colorectal cancer (CRC) screening rates in Federally Qualified Health Centers (FQHCs). Because EBI adoption and implementation is the central goal of implementation science and practice, we begin with our definition of EBIs.

*Evidence-based interventions* (EBIs) include any action or set of actions that delivery systems enact to improve health behaviors, health outcomes, or health-related environments (e.g., built and communication environments that support healthy behaviors). EBIs target factors that directly contribute to health as compared to implementation strategies, which target factors that contribute to EBI adoption, implementation, scale-up, or sustainment. Interventions include what Brown et al. [21] refer to as programs, practices, principles, procedures, products, pills, and policies (the seven Ps). Interventions are evidence-based to the extent that they are supported by research that has established a causal relationship between the intervention and a specified improvement in individual- or population-level health behaviors, health outcomes, or health-related environments.

For colorectal cancer screening, EBIs would include age- and risk-appropriate colorectal cancer screening using either FIT, iFOBT, or colonoscopy, followed by diagnostic screening and treatment as indicated [22].

### Five classes of implementation strategies

#### Dissemination strategies

 include any action or set of actions that target public health and healthcare decision-makers’, clinicians’, and other staffs’ awareness, knowledge, attitudes, and intention to adopt an EBI. Within this classification system, communication strategies that directly target patients or a population with the goal of changing their health-related beliefs, knowledge, or behaviors would be classified as EBIs rather than as dissemination strategies. Dissemination strategies are the primary strategies that synthesis and translation systems use but also are used by actors in both support and delivery systems.

The NIH defines dissemination research as the study of the “targeted distribution of information and intervention materials to a specific public health or clinical practice audience” [23]. Consistent with this definition, dissemination begins with the identification of an intended public health or healthcare audience or audiences (sometimes referred to as audience segmentation) [24]. Identifying audience(s) often is followed by formative research to customize dissemination strategies to fit audience needs and preferences. Dissemination involves two broad categories of strategies: (1) developing messages and materials and (2) distributing EBIs, messages, and materials for a specific audience or audiences.

#### Developing messages and materials

Message development strategies involve framing information about an EBI to persuade the intended decision-making audience to adopt it [25]. Material development strategies include packaging EBI materials into a format that the intended audience can interpret and use [26]. The National Cancer Institute’s “Research Tested Interventions Program,” for example, packages EBIs into a standardized template that includes information on the EBI’s intended population and setting, targeted outcomes, and evidence base and also provides intervention materials and implementation guidance (See https://rtips.cancer.gov/rtips/index.do).

#### Distribution of EBIs, messages, and materials

Distribution strategies focus on ensuring that EBIs, messages, and materials reach intended audiences through a range of distribution channels, including mailings, websites, publications, webinar or in-person presentations, interpersonal connections, and mass media among others. To be most effective, distribution should engage the channels that intended audiences already trust and access for EBI-related information [26, 27]. Diffusion of Innovations Theory identifies interpersonal connections as particularly important to promoting the spread of new innovations [28].

Outcomes used to assess the effectiveness of dissemination strategies include the extent to which EBIs, messages, and materials reach the intended audience(s) and affect their EBI awareness, knowledge, attitude, and intention to adopt. Relevant measures include, for example, Pankratz et al.’s [29] measure of healthcare providers’ perceptions of an intervention.

Dissemination strategies to promote colorectal screening might include formative work to identify who within an FQHC makes the decision to adopt new practices. Findings from formative research with these decision-makers would then be applied to customize messages about the value of CRC screening together with materials about CRC screening EBIs. Messages and materials would then be distributed through channels known to have broad reach to the identified decision-makers such as Primary Care Association conferences, newsletters, or continuing education opportunities.

#### Implementation process strategies

are enacted by those working within delivery systems and pertain to processes or activities that implementation or quality improvement teams perform to plan, select, and integrate an EBI into practice. Processes often are categorized within stages, for example, the stages of exploration, adoption/preparation, implementation, and sustainment [30]. As the name implies, implementation process strategies target the “process” level of the Consolidated Framework for Implementation Research (CFIR) and focus on how well teams execute activities required to select, adapt, and integrate an EBI. Implementation process strategies may begin prior to the selection of an EBI and typically are EBI-agnostic. In other words, the same process strategies that would apply to a clinic-based obesity prevention initiative also would apply to a clinic-based vaccination initiative. Numerous authors have proposed planning stages and recommended the processes required to complete those stages such as assessing the context, engaging key stakeholders, prioritizing goals and objectives, selecting and adapting an EBI to fit the context, evaluating processes and outcomes, and sustaining EBI integration over time [31–33]. Measures of implementation process strategies focus on the extent to which activities or processes within each stage were completed in a high quality and timely manner [34–36].

For CRC screening, implementation process strategies might include FQHC staff (1) prioritizing CRC screening rates as an area in need of improvement; (2) identifying one or more sub-populations of patients with low screening rates; (3) assessing potential determinants of those low rates; (4) selecting EBIs that best fit the identified determinants of low screening rates; (5) selecting integration strategies that target determinants of how well those EBIs will integrate into the FQHC setting; (6) adapting EBIs, integration strategies, and contexts to fit; (7) executing the selected EBIs and integration strategies; and (8) monitoring implementation and intervention effectiveness and making indicated improvements. Of note, the FQHC staff might transfer the process strategies they learned in the CRC screening implementation initiative for use in a future effort to implement another type of EBI.

#### Integration strategies

are delivered by actors within delivery systems and include any action or set of actions that target factors contributing to or impeding the optimal integration of a specific EBI into practice. Whereas implementation processes are relatively EBI-agnostic and include activities prior to selection of a specific EBI, integration strategies are applied to integrate a specific EBI into practice. Integration strategies primarily target determinants at the level of individuals (e.g., motivation, self-efficacy) and inner settings (e.g., leadership engagement, communication) [18]. Strategies include, for example, reminder systems, new care teams, revisions to medical record systems, or new equipment [3]. Evaluations of integration strategy effectiveness ideally would focus on changes in the targeted determinants and also their effects on implementation outcomes. For example, an evaluation of a new care team might assess effects on interdisciplinary coordination—the targeted determinant—in addition to their impact on implementation outcomes (e.g., fidelity, penetration, feasibility) [37].

For CRC screening, integration strategies might include reminder systems that target primary care providers’ awareness of when their patients are due for screening, monitoring those patients’ subsequent screening rates, and providing feedback to primary care providers on those rates.

#### Capacity-building strategies

are delivered by support systems and target individuals’ general capacity (motivation, self-efficacy) to execute implementation process strategies (described above). Capacity-building strategies include training, technical assistance, tools, and opportunities for peer networking, among others [6]. The Institute for Healthcare Improvement, for example, provides strategies to build delivery-system capacity to execute the implementation process strategies that comprise their Model for Improvement [38]. Jacobs et al. [39] provide strategies to build public health delivery system capacity to execute the implementation process strategies described in their planning framework. The outcomes of capacity building strategies focus on individual and collective self-efficacy and motivation to execute implementation process strategies and on their actual completion (extent, quality, timeliness) of those implementation process strategies [6, 35, 36].

For FQHCs, capacity building strategies might involve a support system actor inviting FQHC improvement staff to participate in the Institute for Healthcare Improvement’s online school’s sessions and then providing them with in-person quality improvement coaching sessions as they progress through each implementation process.

#### Scale-up strategies

are enacted by support system actors with the goal of getting multiple settings to implement a specific EBI. Scale-up strategies target determinants at the level of individuals, inner settings, and outer settings. At the individual level, they target motivation, capability, and opportunity [40]. At the inner setting level, they target leadership engagement, resources, and infrastructure among others. At the level of the outer setting, scale-up strategies may target public policy; human and material resources; and cross-setting learning, collaboration, and competition [41]. Examples of scale-up strategies include train-the-trainer initiatives, infrastructure development (supply chains, data systems), quality improvement collaboratives, benchmarking, policy advocacy, and recognition systems [42, 43]. The outcomes of scale-up strategies focus on individual or setting-level changes such as increased motivation or capacity to implement or actual implementation of a specific EBI across multiple settings.

To take CRC screening EBIs to scale, an intermediary organization might employ a staged approach and initially partner with a few FQHCs to develop the combination of EBIs, dissemination strategies, implementation planning strategies, and integration strategies they intend to take to scale (i.e., the change package) [43]. They would then motivate FQHCs’ leadership to adopt the change package and educate staff on how to use it. The intermediary organization might then employ technical assistance and benchmarking to support and sustain FQHCs’ efforts to execute the change package. They may also create a quality improvement collaborative or use other strategies to facilitate peer networking, support, and learning across FQHCs [44].

## Conclusions

Existing taxonomies provide both names and definitions for their lists of implementation strategies. Building on Proctor et al.’s [5] reporting guidelines, the proposed classification system adds greater conceptual clarity to these definitions by classifying strategies according to who enacts them (actor) and the levels and determinants they target (action targets). These distinctions will aid communication among those engaged in both implementation science and practice, who will now be able to identify their need for or use of implementation strategies as aligning with one (or more) of the five classes. Placing strategies into five broad classes will also assist those who are seeking implementation strategies, whether for research or practice. Accordingly, Table 2 provides references for lists of strategies that are applicable to each class. Finally, the classifications will assist systematic reviewers in aggregating findings across implementation strategies by providing an additional scheme for identifying studies with comparable findings.

**Table 2:** Five classifications for implementation strategies

Classification	Category of actor [10]	Action target (determinants and levels)	Example strategies	Outcomes used to assess effectiveness	Example strategy lists/descriptions
Dissemination strategies	All 3 ISF systems	Awareness, attitude, knowledge, and intention to adopt a specific EBI Targets levels of intervention and individual	• Develop EBI messaging, packaging, and pricing customized to audience • Distribute customized EBI messages and packages through channels with optimal reach	Distribution reach to target audience EBI awareness, knowledge, attitude, and intention to adopt	Dearing & Kreuter (2010) [26] Kreuter & Bernhartdt (2009) [27]
Implementation process strategies	Delivery system	How well teams execute activities required to select, adapt, and integrate EBIs generally Targets level of process	• Engage stakeholders • Assess context (need, capacity) • Establish goals and objectives • Select EBI and implementation strategies that fit • Adapt EBI, strategies, and context • Evaluate processes and outcomes	Extent, quality, and timeliness of the completion of activities related to specific implementation process strategies	Aarons et al. (2011) [30] Chinman et al. (2004) [32] Institute for Healthcare Improvement [38] Meyers et al. (2012) [33]
Integration strategies	Delivery system	Factors that facilitate or impede optimal integration of a specific EBI into a specific setting Targets levels of individual and inner setting	For a specific EBI: • Institute reminder systems • Revise professional roles • Provide supervision • Modify medical record systems • Implement tools for quality monitoring	Individuals’ motivation, capability, and opportunity to implement an EBI Team coordination/ communication Fidelity, feasibility, acceptability	Fixsen et al. (2009) [48] Mazza et al. (2013) [3] Powell et al. (2015) [2]
Capacity-building strategies	Support system	Motivation and capability to engage in implementation process strategies (in general, not related to a specific EBI) Targets levels of individual and processes	Across multiple settings: • Training to build general capacity • Technical assistance and facilitation for implementation processes • Tools to support implementation processes	Individual and collective self-efficacy and motivation to engage in implementation process strategies Team completion of implementation process strategies	Leeman et al. (2015) [6] Jacobs et al. (2014) [39] Wandersman et al. (2012) [49]
Scale-up strategies	Support system	Motivation and capacity to integrate a specific EBI into practice Targets level of individual, inner setting, and outer setting	Across multiple settings: • Training to build EBI-specific capacity • Technical assistance and facilitation (EBI specific) • Implementation toolkits • Quality improvement collaboratives • Benchmarking • Recognition systems • Infrastructure development • Changes to fee for service lists/formularies	Motivation and capacity to implement, and actual implementation of an EBI across multiple settings	Barker et al. (2016) [43] Milat et al. (2016) [42]

*ISF* Interactive Systems Framework, *EBI* evidence-based intervention

Consistent with the intent of Proctor et al.’s guidelines, this classification system’s focus on targets of action (levels and determinants) has potential to aid in selecting and strategically combining multiple strategies [45, 46]. For example, by applying intervention mapping methods, implementation strategies could be selected to match determinants across the CFIR levels of the individual, inner setting, and outer setting [47]. By identifying the targeted determinants and measuring strategies’ effects on those determinants, implementation researchers can begin to disentangle the contributions of discrete strategies when multiple strategies are used in combination. They also could advance understanding of the mechanisms through which implementation strategies influence EBI adoption, implementation, scale-up, and sustainment by facilitating the alignment of strategies to theories that explain how they work [46].

This classification is intended to be broadly applicable to EBIs that target changes in individuals, populations, environments, and policy. Although our illustration applied the classification to a clinical EBIs, we easily could have illustrated their applicability to implementation strategies related to policy EBIs (e.g., to regulate tobacco marketing) or to environmental EBIs (e.g., to increase access to healthy foods and beverages).

The next steps for developing this framework might include mapping it to existing taxonomies, such as those developed by Powell et al. [3] and Mazza et al. [4]. Subsequently, a review of the literature and/or Delphi approach might be applied to generate lists of strategies for classes that may be underrepresented in existing taxonomies (e.g., scale-up strategies or dissemination strategies). Further work might also explore the need for additional classes. For example, are different strategies used to de-escalate or to sustain an EBI within a specific setting beyond those that would be listed as integration strategies? Additionally, there may be a need to identify and refine taxonomies of outcomes (like those identified by Proctor et al. [37]) that are relevant for each of these classes (e.g., dissemination, scale-up, etc.).

This proposed classification system reflects the ongoing efforts of implementation scientists to develop clearer and more meaningful ways of communicating about the strategies that are crucial to moving EBIs into real-world practice. Structuring strategies into classes has potential to add and facilitate clearer reporting of implementation research findings, alignment of strategies with relevant theories, synthesis of findings across studies, and identification of potential gaps in current strategy listings. Organizing strategies into classes also will assist users in locating the strategies that best match their needs.

## Abbreviations

CRC: Colorectal cancer
EBI: Evidence-based intervention
FQHC: Federally Qualified Health Center
IHI: Institute for Healthcare Improvement
ISF: Interactive Systems Framework
NIH: National Institutes of Health
